# Technical Note: The development of a multi‐physics simulation tool to estimate the background dose by systemic targeted alpha therapy

**DOI:** 10.1002/mp.14111

**Published:** 2020-03-31

**Authors:** T. Xu, T. Liu, G. Li, C. Dugal, N. A. Aydemir, Y. Liu, J. C. Roeske

**Affiliations:** ^1^ Canadian Nuclear Laboratories Chalk River ON K0J 1J0 Canada; ^2^ Department of Electrical and Computer Engineering Clarkson University Potsdam NY 13699 USA; ^3^ Department of Radiation Oncology Loyola University Medical Center Maywood IL 60153 USA

**Keywords:** alpha‐immuno‐conjugate (AIC), computational fluid dynamics (CFD), Monte Carlo (MC), targeted alpha therapy (TAT)

## Abstract

**Purpose:**

To predict biological effects of targeted alpha therapy (TAT) in preclinical studies, dosimetry calculations based on the micro‐level distributions of emitters are essential. Due to the saturation of the tumor antigenic sites and bonding breaks by decay, some of Alpha‐immuno‐conjugate and decay daughters may inevitably be transported by convection and diffusion along with blood or lymphatic circulation. This results in highly nonuniform and unsteady distributions of irradiation sources. Since the micro‐level distribution of emitters cannot be measured and obtained in patients with current technology, a modeling toolset to give more insight of the internal dose could be an alternative.

**Methods:**

A multi‐physics model based on a Monte Carlo microdosimetry technique and computational fluid dynamics (CFD) modeling was developed and applied to multiple internal irradiation sources. The CFD model tracks the path of the radionuclides and the dose model is capable of evaluating the time‐dependent absorbed dose to the target.

**Results:**

The conceptual model is capable of handling complex nonuniform irradiation sources in vasculature. The results from the simulations indicate that the assumption of homogeneous and motionless distribution of the administered activity used in the conventional dose calculation tends to significantly underestimate or overestimate the absorbed dose to the vascular system in various scenarios.

**Conclusion:**

Modeling the *in vivo* transport of radionuclides has the potential to improve the accuracy of TAT dose estimates. It could be the first step to develop a simulation tool set for assessing absorbed dose to tumor or normal tissues and predict the corresponding biological responses in the future.

## 1 INTRODUCTION

Targeted alpha therapy (TAT) using Alpha‐immuno‐conjugates (AIC) is a promising application of high linear energy transfer radiation to treat various cancers. Targeted alpha therapy can significantly improve drug delivery efficiency by a targeting molecule that fixes onto membrane bound antigens on the surface of the cancer cell.^1^ However, the characterization of the AIC targeted delivery process in the vascular environment is very challenging due to the small scale of AIC particles, decay chain of the labeled radionuclide, and the complex *in vivo* vascular system.^2^ To simplify such a complex system, some optimal conditions have been utilized in dose models, assuming homogeneous and stationary distribution of irradiation sources, available antigenic sites in the tumor to the monoclonal antibodies (mAbs), etc*.* ^3^ This actually does not reflect the real situation *in vivo.* For example, McDevitt et al.^4^ reported for a specific activity of 3.2 MBq/mg of conjugate containing ^225^Ac‐huM195, the labeling rate is 1:1000. This means that the unlabeled mAbs containing 99.9% of total antibody would compete for binding the antigenic sites with the remaining 0.1% labeled mAb. Consequently, the saturation of antigenic sites blocks the access of labeled mAbs to the tumor surface. When a radionuclide is attached to a protein, for example, antibody, it has its chemical properties that allow it to be bonded. When the radionuclide decays, the recoil energies and different chemical properties of its daughter that is still an emitter may make the protein bond unstable and broken. These free AICs and daughter decay products may be transported by convection and diffusion along with blood or lymphatic circulation causing spatial and temporal variation in transport. The homogeneous assumption on the AIC distributions in tissues and organs may not adequately reflect the biological effects under the situation of strong convection. Hence, the mean absorbed dose to the sensitive volume using the dosimetry formula described in MIRD Pamphlet 21^5^ is not expected to accurately predict the biological responses of TAT.^6^

Computational fluid dynamics (CFD) is a numerical method of solving fluid mechanics problems based on the Navier–Stokes equations, which include a series of time‐dependent partial differential equations describing conservation of mass, momentum, and energy for a viscous fluid.^7^ Computational fluid dynamics is an efficient method to study blood flow behavior by modeling fluid flow through numerical simulations. To model the background dose under convection and diffusion, an earlier study by the authors^8^ established a multi‐physics‐based approach for modeling single AIC continuum delivery in a blood vessel at the mesoscale. The mesoscale simulation that tracked the single AIC in a vascular capillary has been shown to sufficiently resolve the near‐field AIC‐attached flows. Based on the work related to *in vivo* nuclear medicine transport due to the blood circulation described in Ref. [8], the history of the trajectories of AIC with radionuclide installed can be tracked. This provides the required input of irradiation source distributions for the Monte Carlo (MC) radiation transport code such as Geant4^10^ to evaluate the absorbed dose on the target cell. However, this investigation is only limited to the interaction between single AIC and incompressible Newtonian flow. A few demonstration examples based on this restriction have been presented in Ref. [8].

Nevertheless, trillions of radionuclides could be used in a clinical trial to create an effective activity to eradicate cancer cells. For example, in a melanoma clinical trial,^11^ the administered conjugate contained up to 25 mCi of ^213^Bi with a specific activity of 3.2 mCi/mg. The administrated conjugate comprises both radiolabeled and unlabeled monoclonal antibody. The number of ^213^Bi atoms in 3.2 mCi (1.184 × 10^8^ Bq) for 1 mg of the administered conjugate can be given by Ref. [11]:

(1) $$N=\frac{A}{\lambda }=4.67\times 10^{11}$$

where *A* is activity (1.184 × 10⁸ Bq in this case) and *λ* is the decay constant which is given by,

(2) $$\lambda =\frac{\ln\left(2\right)}{T_{1}/2}$$

In Eq. (2), T_½_ is the half‐life of the selected radionuclide, which is 46 min^13^ for ^213^Bi.

To create the same activity for the long‐lived radionuclide ^225^Ac, the number of atoms in 3.2 mCi (1.184 × 10^8^ Bq) in 1 mg of the administered conjugate can be as high as 1.47 × 10^14^. Multiple particles in the blood flow involve particle–particle interactions. Those interactions may consist of AIC–AIC collisions, AIC–RBC (red blood cell) collisions and AIC–fluid interactions. AIC–RBC interactions are not considered currently. To this end, modeling the path of a single particle only forms the basis to analyze the transient dose in TAT clinical trial. To model the transport of such a large number of atoms in preclinical studies and predict the background dose to the normal cells, the single AIC continuum delivery model was extended to a multiple particle model that includes the AIC collision model and multiple immersed boundaries model. The collision model to accommodate all the interactions is required for the analysis of AIC delivery in clinical trials. Therefore, in this work, a mathematical model to consider multiple AICs transport in a capillary has been developed. To the best of our knowledge, this work is the first one to propose a comprehensive model which includes governing equations, particle tracking, collision model, and microdosimetry calculators.

In order to analyze the efficacy and toxicity with significant stochastic variations in the energy deposited within the cellular nucleus, modern clinical trials and preclinical studies require increasingly large amounts of data from complex and computationally intensive microdosimetric modeling to plan a safe and effective treatment based on the interpretation of biological endpoints. To be practical for preclinical studies, the analysis must be completed within an affordable amount of time. Due to the large computational time required for detailed physics models, microdosimetric modeling using the Monte Carlo method becomes very expensive, and in some cases, it is not clinically practical.

The Monte Carlo method is a sampling method used to imitate real life and to make predictions by using random numbers to generate probabilities. Roeske and Hoggarth^14^ used a simplified method to perform MC simulations to estimate the single‐hit spectra of alpha particle transport with an interpolating polynomial fitted from the range–energy data. As a result, the distribution of path length, average energy deposited, and specific energy for a single alpha particle traversal through the cell nucleus can be determined. It is therefore simple and efficient when compared against other MC simulations using more sophisticated models.^11^, ^15^ For a single alpha particle simulation with 50 000 samples, Roeske and Hoggarth's method takes no more than a few seconds to perform one calculation. To estimate the spread of the spectra made by multiple irradiation sources due to the heterogeneous distribution of AICs and decay daughters in transport, an extension to the original model is essential.

**Fig. 1:**
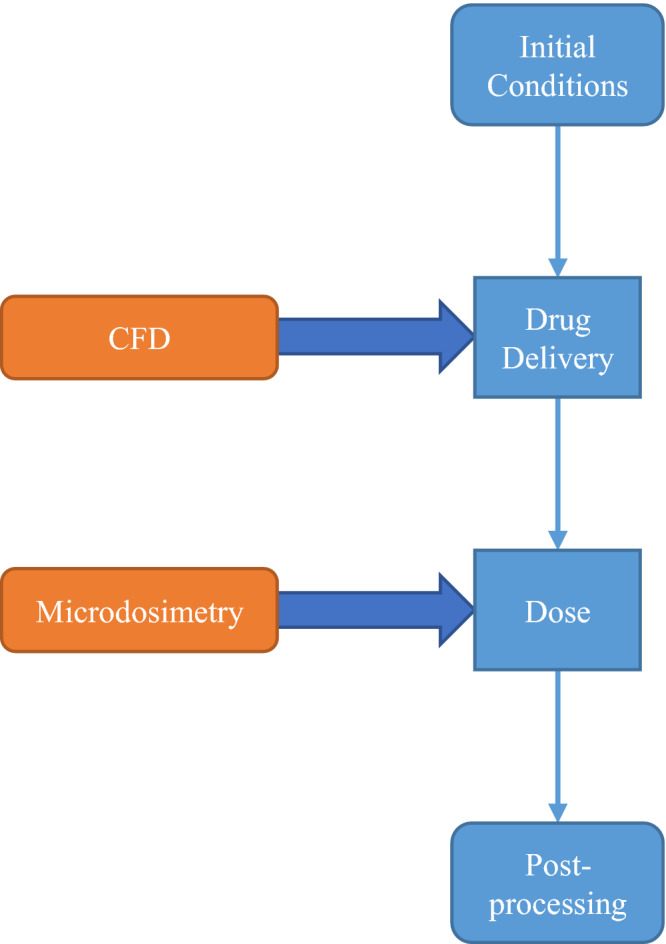
The processes of the multi‐physics approach for the internal dose estimation. [Color figure can be viewed at wileyonlinelibrary.com]

Preclinical dose response studies have shown that the short range of alpha particles relative to the scale of human organ dimensions and associated target volumes can lead to a highly nonuniform irradiation distribution in the target.^6^ ^.^ With the coupling of a Computational Fluid Dynamics (CFD) model, a decay scheme, and a microdosimetric model illustrated in Fig. 1, the transient and nonuniform behavior of the background dose caused by the transport of the multiple AICs in vasculature can be investigated in detailed manner. This paper mathematically models the integrated process of (a) the AIC transport resulting in drug distributions under vasculature, and (b) the microdosimetry analysis based on the calculated micro‐level AIC distributions. This framework was established with the goal of improving the understanding of the efficacy and toxicity in humans or live animals. The development of such a model can potentially be used for personalized patient treatments and dose schedule in the future.

## 2 MATERIALS AND METHODS

### 2.A Hemodynamic model for multiple AIC transport

The single AIC transport model^8^ was extended to establish a solution strategy for mesoscale modeling of the time‐dependent micro‐level distributions of multiple AICs in a blood capillary.

In such an extended model, the following two‐dimensional (2D) governing equations including conservation equations for mass and momentum for incompressible Newtonian fluid flows have been used,

(3) $$\begin{array}{c} \vec{\nabla }\cdot \left(\rho \vec{V}\right)=0\\ \frac{\partial }{\partial t}\left(\rho u\right)+\vec{\nabla }\cdot \left(\rho \vec{V}u\right)=\vec{\nabla }\cdot \left(\mu \vec{V}u\right)-\frac{\partial p}{\partial x}\\ \frac{\partial }{\partial t}\left(\rho v\right)+\vec{\nabla }\cdot \left(\rho \vec{V}v\right)=\vec{\nabla }\cdot \left(\mu \vec{\mathrm{Vv}}\mu \right)-\frac{\partial p}{\partial y} \end{array}$$

In Eq. (3), u and v are Cartesian components of the velocity vector $\vec{V}$. The variables µ and ρ are viscosity and density, respectively, and p is pressure. Since the isentropic assumption is used, energy conservation is not considered.

An existing Euler‐implicit and finite‐volume flow solver based on a pressure correction scheme is utilized. Note that finite‐volume method is a method for representing and evaluating partial differential equations in the form of algebraic equations. The detailed methodology can be found in Ref. [12]. The discrete AICs in the blood are modeled by the Ghost Cell Immersed Boundary Method (GCIBM), originally proposed by Charles Peskin.^9^ The following is the procedures to implement GCIBM and simulate moving AICs on a Cartesian mesh in a 2D channel. The velocity vector at solid boundaries is split into its tangential and normal components ($u_{B}^{t}$,$u_{B}^{n}$). Hence, $U_{B}=u_{B}^{t}e^{t}+u_{B}^{n}e^{n}$ (where $e^{t}$ and $e^{n}$ are the unit vectors tangential and normal to the walls, respectively) satisfying both the “slip condition” and the “impermeability condition.”

(4) $$\frac{\partial u_{B}^{t}}{\partial n}=0≅\frac{u_{I}^{t}-u_{G}^{t}}{\Delta n}$$

(5) $$u_{B}^{n}=0≅\frac{1}{2}\left(u_{G}^{n}+u_{I}^{n}\right)$$

The subscripts “*G*” and “*I”* in Eqs. (4) and (5) denote the value at the “ghost” and “image” locations, respectively. The detailed implementation is described in Ref. [8]. Following the same methodology, the Immersed Boundary Method for multiple particles has been developed and integrated into the flow solver. Extensive tests against various benchmark cases for a single and multiple particles were made.^8^ Since more than 10^12^ radionuclides could be used in a clinical trial to create an effective activity to kill cancer cells, all the particles may move at different velocities, and fast‐ and slow‐moving particles may potentially collide. A multiparticle collision model is crucial in the investigation of mechanisms for clustering and agglomeration of particles and the formation of particle agglomerates in a dense solid particle flow. Such a collision model, formulated in Eq.  (6), is developed and extended to model multiple particle interactions. Those interactions may consist of AIC–AIC collisions, AIC–RBC (Red Blood Cell) collisions, and AIC–fluid interactions.^11^

(6) $$\begin{array}{cc} {\vec{u}}_{p,1}^{{}^{\prime }}\;=\; & {\vec{u}}_{p,1}-\frac{m^{\text{eff}}}{m_{p,1}}\left(1+\varepsilon \right)\left({\vec{u}}_{p,12}\cdot \vec{e}\right)\vec{e}\\ {\vec{u}}_{p,2}^{{}^{\prime }}\;=\; & {\vec{u}}_{p,2}-\frac{m^{\text{eff}}}{m_{p,2}}\left(1+\varepsilon \right)\left({\vec{u}}_{p,12}\cdot \vec{e}\right)\vec{e} \end{array}$$

In Eq. (6), *ε* is the restitution coefficient. The velocity vector with the superscript of prime stands for the post‐collision velocity vector. Note that for *ε* = 1, the total kinetic energy is conserved, and the collision occurs elastically; whereas, for *ε* = 0, some kinetic energy is lost, and the collision occurs non‐elastically. The unit vector $\vec{e}$ between the two particles is defined as,

(7) $$\vec{e}=\frac{{\vec{r}}_{1}-{\vec{r}}_{2}}{\left|{\vec{r}}_{1}-{\vec{r}}_{2}\right|}=\frac{{\vec{r}}_{12}}{\left|{\vec{r}}_{12}\right|}$$

where ${\vec{r}}_{12}$ is the vector which joins the centers of particles when they come in contact.

The relative velocity of two particles before collision is:

(8) $${\vec{u}}_{p,12}={\vec{u}}_{p,1}-{\vec{u}}_{p,2}$$

The effective mass is defined as:

(9) $$m^{\text{eff}}=\frac{m_{p,1}\cdot m_{p,2}}{m_{p,1}+m_{p,2}}$$

### 2.B Multiple alpha particle traversal microdosimetric model

Following Roeske and Hoggarth's^14^ single‐hit MC simulation methodology, a microdosimetric model for multiple irradiation sources was developed in Fortran programming language, which includes the modeling of a multiple moving sources on the surface of the target cell and outside of the target cell configurations. It has been integrated into an in‐house code APLIT (alpha particle location in transit)^8^ to calculate the deposited energy based on the spatial and temporal distributions of various emitters and target pairs. The Multiple Source Microdosimetric model reads in the all irradiation source locations output from the CFD model at each time step. Due to the aggregation, some AICs are assumed to form agglomerates and clusters as irradiation sources to the target. The time‐dependant distance between the irradiation sources and the target nucleus can be subsequently determined. As a result, the first and second moment of single‐hit specific energy distribution <$z_{1}$> and <$z_{1}^{2}$> can be calculated, respectively.^14^ The average specific energy <z> for multi‐hit distribution by one source configuration is given by Ref. [16, 17],

(10) $$〈z〉=〈n〉〈z_{1}〉,$$

and the corresponding variance can be derived as Ref. [16],

(11) $$\sigma^{2}=〈n〉〈z_{1}^{2}〉,$$

where n is the average number of hits from the single source containing multiple AICs. In a Monte Carlo implementation, it is determined by the multiplication of the probability of the hits on the target and the cumulated activity at the given time window. The other option is to use the probability mass function of the Poisson distribution directly to estimate the average hits without the computational intensive MC sampling. The first and second moments of two combining multihit were investigated and derived in Ref. [16]. Following the fundamental theorem in microdosimetry, the similar relations of the average specific energy at time t for multiple moving source configurations can be given by the following summation of all the sources,

(12) $$〈z_{t}〉=\int_{0}^{t}\sum_{i=1}^{i=m}〈n^{i}〉〈z_{1}^{i}〉dt,$$

where $〈n^{i}〉$ is the average hits from the ith source and $〈z_{1}^{i}〉$ is the average specific energy of the single‐hit distribution by the ith source. The value m is the total number of irradiation sources. Similarly, the total variance at the time t can be determined by,

(13) $$〈\sigma_{t}^{2}〉=\int_{0}^{t}\sum_{i=1}^{i=m}〈n^{i}〉〈{\left(z_{1}^{i}\right)}^{2}〉dt,$$

where ${\left(z_{1}^{i}\right)}^{2}$ is the second moment of the single‐hit distribution by the ith source.

A single moving irradiation source has been simulated using the code APLIT and Geant4 as a verification. The excellent agreement for the mean specific energy is obtained, which calculates the average dose per hit on a cell nucleus with 5‐μm long in radius. It is an isotropic point alpha source with 5.8 MeV energy, moving from (−15, 0, 0) in μm initially to (15, 0, 0) in μm. The physics model used in Geant4 is the emstandard_top3 package, described as “designed for any applications required higher accuracy of electrons, hadrons and ion tracking without magnetic field.” ^18^ The statistical uncertainty of these results is smaller than the size of data points. The difference between the results provided by Geant4 and by APLIT is within 1%.

To investigate a radionuclide with a long decay chain, a continuous decay model with approximate branching and approximate alpha decay energies was developed. The expected radionuclide populations as a function of time were provided to the multiple source dosimetric models.

### 2.C Methodology used in the integration tests

The APLIT code has integrated the above‐described CFD model, the microdosimetric model, and decay model to produce coupled simulation results. The code reads the input, geometry, and mesh file that contain the control parameters initially. Then, the immersed boundary module is called and the CFD module with pressure and velocity components solver is invoked. If the convergence criteria are not met (residual of ≤0.5 × 10⁻⁶), the code will switch back to the immersed boundary module. After the convergent results were obtained by the CFD module, an inner loop containing the decay scheme and microdosimetric module was used to calculate the cumulated activity, dose, and variance. The code will proceed to the next time step as long as the AICs still remain in the computational domain; otherwise, the simulation ends.

The integration test case was designed based on a melanoma clinical trial. In the melanoma clinical trial by Allen et al.,^11^ the administered conjugate contained up to 25 mCi of ^213^Bi with a specific activity of 3.2 mCi/mg. The number of ^213^Bi atoms in 1 mg conjugate with specific activity of 1.184 × 10⁸ Bq/mg is $4.67\times 10^{11}$. The same specific activity and emitters in the melanoma clinical trial were used as APLIT inputs so that a comparison with experimental results can be made.

The capillary model selected in this test is made up of a single endothelial cell (EC) that wraps around to form the capillary wall, butting up to contiguous capillaries. The administered AICs are intended to move along with the blood circulation in the capillary lumen, killing tumor cells in the perivascular. In the case of melanoma, the AIC targeted both perivascular cancer cells and pericytes that express the same antigen. As a result, those cells will receive a high dose of alpha irradiation which may induce a biological effect.

In this integration test, only the perivascular nucleus was investigated as a sensitive volume. As a reasonable approximation, both the endothelial and perivascular cells were modeled as being made solely of water.^19^

Mesoscale modeling has been conducted to gain an understanding of the following aspects: (a) Dynamics of AIC‐attached blood flow interaction in vasculature, (b) influence on AIC motion in the main flow direction and the perturbations/instabilities of the AIC trajectories in the transverse direction and (c) particle–particle interactions in multiple AIC‐attached flow.

**Fig. 2:**
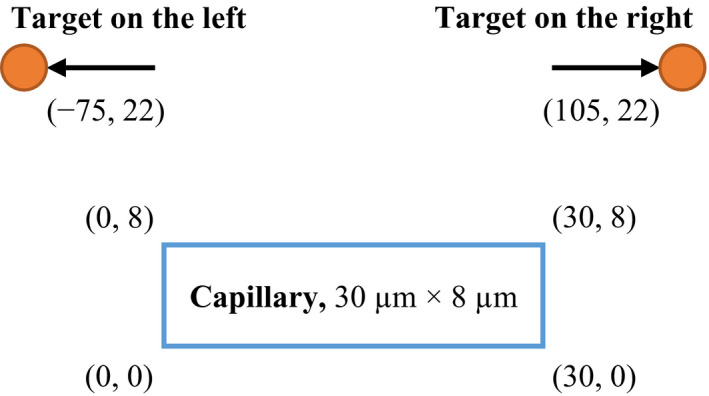
Relative position of the target and the capillary domain in the integration tests. [Color figure can be viewed at wileyonlinelibrary.com]

The effect of toxicity on healthy cells or the efficacy to kill tumor cells during the multiple AICs transport process can be evaluated when coupled with the Multiple Source Microdosimetric model in APLIT. In the integration mesoscale test for the endothelial cell (EC) capillary domain, the following test settings were used:

- A 30‐µm long and 8‐µm wide 2D rectangular computational domain is used for the CFD model in the two‐dimensional domain (a typical EC geometry) with a grid of 750 × 200 cells. A Dirichlet boundary condition is specified for the inlet where the inlet velocity at the left side of the computational domain is given as 0.03 cm/s and a Neumann boundary condition is imposed for the outlet boundary. The top and bottom boundaries are impermeable and the velocities are specified to be 0.0 cm/s.
- The fluid is assumed to be an incompressible, Newtonian and buoyancy and other contributions to the source term have also been considered to be negligible. The blood density is assumed to be 1.025 g/cm^3^ and the viscosity is fixed at 4.0 × 10⁻^3^ Pa s.^20^
- Although the size of AICs is on the order of magnitude of nanometers, the AICs are assumed to be aggregated in the range of 0.2 µm in radius and will not be separated and deformable in the life of simulation for simplicity. The assumption is based on the fact that some polymersomes of the same size may be potentially used as the carriers of emitters.^21^
- The alpha particle energy is 8.3 MeV emitted by ^213^Bi.^5^ The 50 000 samples^16^ are used to evaluate the corresponding single hit average dose and variance.
- The radius of Perivascular cell and nucleus radius is 5 and 4 μm, respectively. The origin of the coordinate is located at the bottom left of the domain. The target cell is at the left‐hand side of the capillary (*X*_0_ = −75 μm, *Y*_0_ = 22 μm) and at the right‐hand side (*X*_0_ = 105 μm, *Y*_0_ = 22 μm), respectively. The relative position between the target and the capillary domain is illustrated in Fig. 2. The unit of the position used in the illustration is in μm.
- The total simulation time is 0.1 s with the time interval of 1.0 × 10⁻^5^ s throughout the simulation.

In this paper, AICs were assumed to be distributed into 158 predetermined source locations in the computational domain and kept in carriers throughout the simulation. The scenarios for particle transport with blood convection and stationary sources were considered and compared. The assumed complex source configuration is to reflect the heterogeneous *in vivo* behaviors of AICs under vasculature. Actually, the nonuniform and unsteady distributions of emitters have been observed in the isolated CFD test shown in Fig. 5 of Section 3.

Since the goal of this work is to investigate the micro‐level distribution of irradiation sources in space and the resulting biological effects, the outcomes with the assumed source configuration will give more insight into the absorbed dose to the target. The tracking of individual emitters is very time consuming and can be performed in the future after the code is parallelized. At the beginning of the simulation, the particles are stationary when they are released at time *t* = 0 s. The first irradiation source is centered at *x* = 7.25 µm and *y* = 4.0 µm. The remainder of the sources are randomly placed from 3 to 25 µm in x direction and from 1 to 7 µm in y direction in the domain of interest. For numerical treatment purposes, the minimal gap between sources is predetermined to be 0.1 µm. After the particles are released when the simulation starts, they are subsequently accelerated up to the velocity of the surrounding fluid by convection until they exit the domain.

As discussed previously, the specific activity (1.184 × 10⁸ Bq/mg) administered in the clinical trial was used in the simulation. For a given 30‐µm long and 8‐µm wide domain, it contains an equivalent mass of 1.5 ×10⁻⁶ mg. As a result, the total administered activity injected in the capillary lumen is about 1.79 × 10^2^ Bq. For individual sources (158 in total), the activity is 1.13 Bq. The total number of ^213^Bi atoms for computation based on Eq. (1) is 7.05 × 10^5^. Based on this setting, the cumulated activity in the total simulation time of 0.1 s in the domain of interest can be as high as 17.9 Bq.s if all emitters are motionless. It is expected the cumulated activity would be supposed to lower than 17.9 Bq.s if some emitters leave the domain and vice versa.

For each time step in APLIT, the hemodynamic data predicted by the CFD module and the activity determined by the decay scheme were obtained and saved. Subsequently, the data are passed to the microdosimetric model to evaluate the specific energy of the target nucleus, average number of hits, and the variance of the dose. In the future, this information can be sent during runtime to the microdosimetry model to achieve the best computational performance.

## 3 RESULTS

**Fig. 3:**
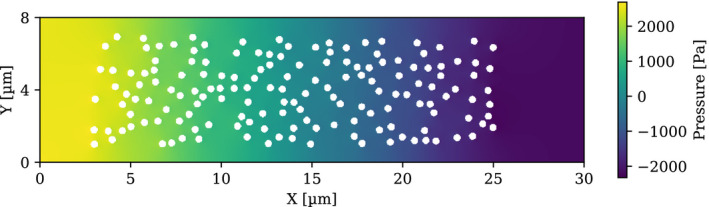
Irradiation source distribution at 0.0 s of the alpha particle location in transit simulation. [Color figure can be viewed at wileyonlinelibrary.com]

**Fig. 4:**
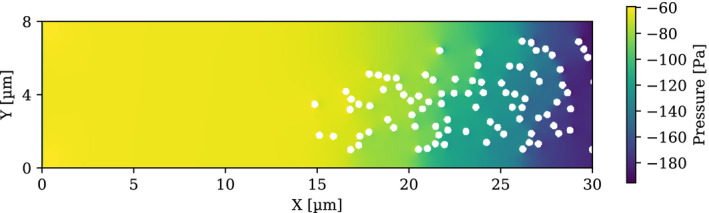
Alpha‐immuno‐conjugate distributions at 0.05 s of the alpha particle location in transit simulation. [Color figure can be viewed at wileyonlinelibrary.com]

The simulation of the test cases described in Section 2.C was performed. Figure 3 shows the irradiation source distribution and the pressure contour for the domain of interest at time 0.0 s, immediately after all the particles were released. It can be seen that all the 158 irradiation sources are in the domain. The source distribution and the static pressure contour at time 0.05 s can be seen in Fig. 4. The color scheme from red, yellow to blue in the contour plot represents the high to low pressure, which is endowed with information on the pressure gradients by which the flow and the irradiation sources were driven. In the future, it can be helpful to evaluate the strength of the chemical bonding when the stress was applied to the bonds and alpha particle was emitted.

**Fig. 5:**
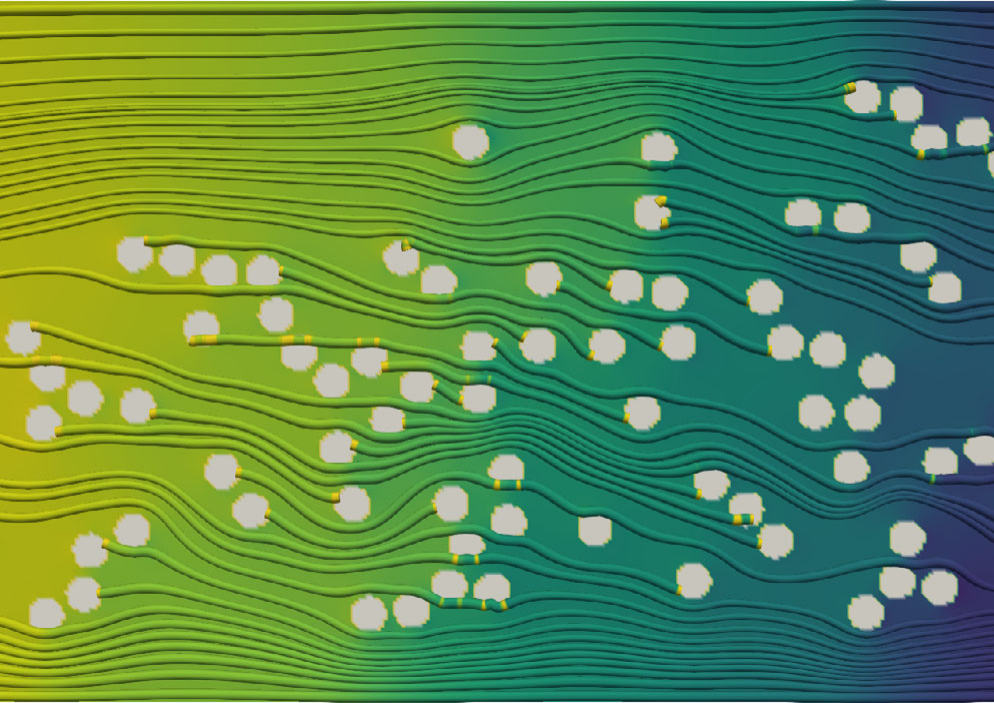
Zoomed‐in velocity vectors and pressure contours at 0.05 s. [Color figure can be viewed at wileyonlinelibrary.com]

Figure 5 is the zoomed‐in view for the simulation at 0.05 s. Note that only portion of the domain from 16.3 to 27.6 µm in X coordinate and from 0 to 8 µm in Y coordinate is displayed. It can be observed that many irradiation sources tend to aggregate and agglomerates and clusters are formed. The main stream flow is observed to move around some irradiation sources. Due to the strong flow resistance of the sources and the boundaries, the flow velocity inside the cluster is much slower than the surrounding flow. Similar phenomena were found in the isolated CFD test. The micro‐level distributions remained nonuniform and changing with time because of the unsteady behavior of the mean flow and the complex interactions among particles.

The previous assumption that the administered conjugate under vasculature is homogeneously distributed could be unrealistic based on the simulation results. Some sources exit the computational domain from 0.05 to 0.1 s. Those sources were assumed to keep the same speed as they left the domain.

The tracking of emitters in the CFD module defines the changing irradiation source configuration with time. Note that not all the individual emitters were tracked. Actually only the assumed 158 irradiation sources with unique positions were monitored for simplification purpose. The AIC contained in the irradiation sources was assumed to move at the same speed with the sources.

**Fig. 6:**
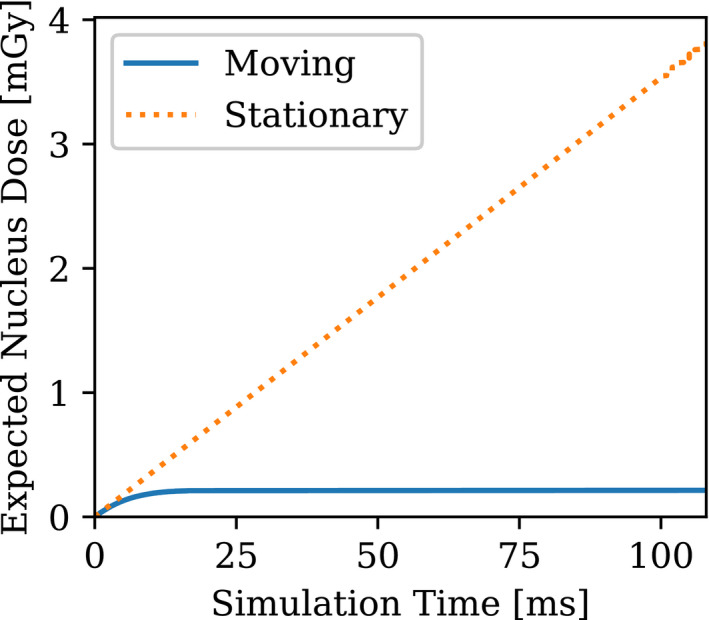
Cumulated dose of alpha particle location in transit simulation vs stationary particles: emitters move out of alpha range. [Color figure can be viewed at wileyonlinelibrary.com]

**Fig. 7:**
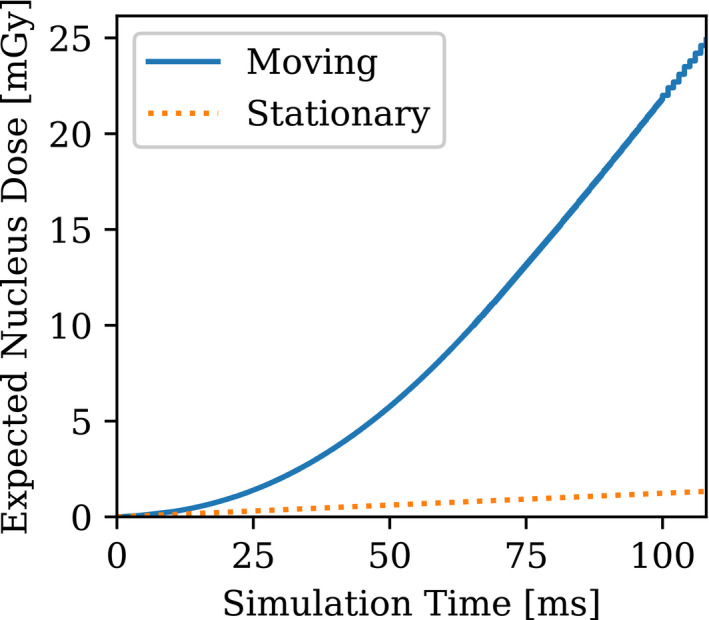
Cumulated dose of alpha particle location in transit simulation vs stationary particles: emitters move into alpha range. [Color figure can be viewed at wileyonlinelibrary.com]

Based on the micro‐level distribution predicted by the CFD module, the histories of the total specific energy calculated by the microdosimetric model are presented in Figs. 6, 7. These figures display the cumulated specific energy deposited on the target over the simulation time based on the theorem formulated in Eq. (12). A sampling of 50 000 was used for each location of sources. This means that 158 average single‐hit doses and the corresponding variances have been evaluated. Multiplied by the average number of alpha hits to the sensitive volume and summed up, the cumulated dose and the total variance at a given time for all the irradiation sources can subsequently be estimated, respectively. Here, the cumulated dose was defined as the total deposited specific energy from the beginning of the simulation to a given time. For the test case shown in Fig. 6, the target cell center is placed at the left‐hand side of the capillary domain at (*X*_0_ = −75 μm, *Y*_0_ = 22 μm) and is not in the computational domain shown in Fig. 2. The moving AICs tend to transport away from the target when the simulation started. As most of the AICs travel beyond the range of alpha particles at time = 0.01 s, the cumulated dose becomes constant as no more hits were experienced by the target nucleus.

A comparison is made between the transport simulations and the stationary alpha particles simulation, assuming the source configuration of the stationary particles is the same at the onset of the simulation shown in Fig. 6. The discrepancy between the moving and stationary sources becomes larger as time increases.

For the test case shown in Fig. 7 when the target cell center is placed at the right‐hand side of the capillary domain at (*X*_0_ = 105 μm, *Y*_0_ = 22 μm) and is not in the computational domain shown in Fig. 2, the average distance from AIC to the center of the sensitive volume is getting closer with time. When most AICs move into the range of alpha particles at time = 0.02 s, the cumulated dose increased significantly because the target had more hits by the stoppers. When some of the stoppers become crossers, the pace of the dose deposited decreases at approximately 0.1 s. A comparison calculation to the stationary particles was also made.

The resulting values of <z> and the corresponding total dose variance $〈\sigma_{t}^{2}〉$ at time = 0.1 s are summarized in Table I. When the sensitive volume is centered at (X_0_ = −75 μm, Y_0_ = 22 μm) or (X_0_ = 105 μm, Y_0_ = 22 μm), both the dose and the variance are of different magnitude. The rule of thumb is that the discrepancies were found to be much higher if the flow direction is unfavorable: that is, when the flow tends to convect emitters away from the target, the stationary sources assumption tends to significantly overestimate the dose. On the contrary, when the flow tends to drive emitters closer to the target, the stationary sources assumption tends to underestimate the dose.

**Table I:** Values of <z> and $<\sigma_{t}^{2}>$ for the multiple moving or stationary sources at time = 0.1 s.

Center of the sensitive volume	(X_0_ = −75 μm, Y_0_ = 22 μm) moving	(X_0_ = 105 μm, Y_0_ = 22 μm) moving	(X_0_ = −75 μm, Y_0_ = 22 μm) stationary	(X_0_ = 105 μm, Y_0_ = 22 μm) stationary
<z> (Gy)	0.00021	0.025	0.0038	0.0013
$<\sigma_{t}^{2}>$ $({Gy}^{2}$)	0.58e‐4	0.0136	1.42e‐3	3.2e‐4

## 4 DISCUSSION

The preliminary mesoscale simulation reveals that when emitters approach the sensitive volume, no matter whether alpha particles are stoppers or crossers, the deposited energy is significantly greater than the contribution by the assumed stationary emitters and vice versa. The discrepancy of the specific energy in the target between moving and stationary setting is significant, demonstrating that the established model is potentially capable of providing a more accurate prediction of internal dosimetry for alpha emitter therapy than the conventional methods. The model is still at an early stage of development. The limitations are: (a) the CFD model was restricted to the two‐dimensional domain; (b) a Neumann boundary condition is imposed for the outlet boundary. The top and bottom boundaries are impermeable; (c) the fluid is assumed to be an incompressible, Newtonian and buoyancy and other contributions to the source term have also been considered to be negligible; (d) similar to some carriers, the AICs are assumed to be aggregated in the range of 0.2 µm in radius and will not be separated and deformable in the life of simulation for simplicity; (e) all properties of cells and blood flow were considered as water equivalent. Some of the individual model validations or verifications were done. A GEANT4 comparison test for the verification of microdosimetric model described in Section 2.B. More validation at three‐dimensional (3D) level can be made to compare the published data after the 2D model is extended to 3D.

Since the 2D or 3D particle tracking is computationally intensive even at the microscopic level, a one‐dimensional blood network idealization is being developed to simplify the particle transport simulation and predict the emitter distributions. Code parallelization and GPU acceleration are also being considered to improve computational performance. Since the evaluation of toxicity is essential in cancer therapy, the accurate estimation of the absorbed dose in vivo is the key to further assess the biological response in clinical studies. The multi‐physics methodology implemented here can track the radioactive sources and more accurately predict the energy deposited in the target cells.

Future work toward developing patient‐specific dosimetry based on the methodology will provide additional insights into the biological response for preclinical studies. To validate a realistic 3D model, some human or animal image‐based data collection can be utilized.

## 5 CONCLUSIONS

This work is a first attempt to establish a computational multi‐physics model to predict the microdosimetry quantities for internally and heterogeneously distributed AICs under convection. Since the micro‐level distribution of emitters is not generally measurable in humans, the combination of the CFD and microdosimetry models could potentially provide a more accurate alternative means of assessing internal dose levels in preclinical studies.

## CONFLICT OF INTEREST

Dr. J. Roeske receives support from Varian Medical Systems, not related to this project. Other authors have no relevant conflicts of interest to disclose.
